# Prognostic Value of Fas/Fas Ligand Expression on Circulating Tumor Cells (CTCs) and Immune Cells in the Peripheral Blood of Patients with Metastatic Breast Cancer

**DOI:** 10.3390/cancers16172927

**Published:** 2024-08-23

**Authors:** Maria A. Papadaki, Eleni Papadaki, Sofia Chatziavraam, Despoina Aggouraki, Kleita Michaelidou, Charalampos Fotsitzoudis, Maria Vassilakopoulou, Dimitrios Mavroudis, Sofia Agelaki

**Affiliations:** 1 Laboratory of Translational Oncology, Medical School, University of Crete, Heraklion, 70013 Crete, Greece; helenipapa@hotmail.com (E.P.); bio3457@edu.biology.uoc.gr (S.C.); daggouraki@yahoo.co.uk (D.A.); k.michailidou@uoc.gr (K.M.); cfotsitzoudis@pagni.gr (C.F.); marvasila@uoc.gr (M.V.); mavroudis@uoc.gr (D.M.); agelaki@uoc.gr (S.A.); 2 Department of Medical Oncology, University General Hospital of Heraklion, 71500 Heraklion, Greece

**Keywords:** Fas (CD95/APO-1), Fas ligand (FasL/CD95L), Fas/FasL pathway, circulating tumor cells (CTCs), peripheral blood mononuclear cells (PBMCs), peripheral immune response, liquid biopsy, breast cancer, biomarkers

## Abstract

**Simple Summary:**

The Fas/Fas ligand (FasL) system is an apoptosis-regulating pathway that holds a key role in tumor immune surveillance and metastasis. Although this pathway has been extensively investigated in pre-clinical models and in primary tumor tissues, however its role in the periphery is largely unexplored. To this end, the expression of Fas/FasL was for the first time assessed on circulating tumor cells (CTCs) and matched peripheral blood mononuclear cells (PBMCs) in the peripheral blood of patients with metastatic breast cancer (BC). The results show that Fas and FasL are frequently expressed on BC patients’ CTCs, with Fas/FasL-co-expressing CTCs emerging as a poor prognostic marker predicting a high risk for disease progression. On the other hand, Fas and FasL were also frequently expressed on BC patients’ PBMCs, and Fas/FasL co-expression on PBMCs emerged as a favorable prognostic marker associated with a low risk for death. These results show that the assessment of Fas/FasL expression on CTCs and PBMCs can provide valuable prognostic information for patients with metastatic BC, and highlight their potential role in the peripheral immune response and metastatic progression of BC.

**Abstract:**

The Fas/Fas ligand (FasL) system is a major apoptosis-regulating pathway with a key role in tumor immune surveillance and metastasis. The expression of Fas/FasL on mammary tumor tissues holds prognostic value for breast cancer (BC) patients. We herein assessed Fas/FasL expression on circulating tumor cells (CTCs) and matched peripheral blood mononuclear cells (PBMCs) from 98 patients with metastatic BC receiving first-line treatment. Fas+, FasL+, and Fas+/FasL+ CTCs were identified in 88.5%, 92.3%, and 84.6% of CTC-positive patients, respectively. In addition, Fas+/FasL+, Fas-/FasL+, and Fas-/FasL- PBMCs were identified in 70.3%, 24.2%, and 5.5% of patients, respectively. A reduced progression-free survival (PFS) was revealed among CTC-positive patients (median PFS: 9.5 versus 13.4 months; *p* = 0.004), and specifically among those harboring Fas+/FasL+ CTCs (median PFS: 9.5 vs. 13.4 months; *p* = 0.009). On the other hand, an increased overall survival (OS) was demonstrated among patients with Fas+/FasL+ PBMCs rather than those with Fas-/FasL+ and Fas-/FasL- PBMCs (median OS: 35.7 vs. 25.9 vs. 14.4 months, respectively; *p* = 0.008). These data provide for the first time evidence on Fas/FasL expression on CTCs and PBMCs with significant prognostic value for patients with metastatic BC, thus highlighting the role of the Fas/FasL system in the peripheral immune response and metastatic progression of BC.

## 1. Introduction

The immune system plays a fundamental role in the control of tumor growth by interacting intimately and continuously with cancer cells within the tumor microenvironment (TME). Immune cells, under normal conditions, can identify and destroy tumor cells; however, in the presence of the tumor, they can be influenced by different factors to act as bystanders or supporters of the tumor, a process named cancer immunoediting. On the other hand, cancer cells can also hijack the mechanisms of immune checkpoint control to avoid the detection and elimination by immune cells and, at the same time, to induce immune cell deregulation and deactivation. Thus, the tumor immune crosstalk is a complex, dynamic, two-directional process [1,2]. Immunotherapy, which aims to restore the antitumor immune responses, has revolutionized the treatment of several cancers including breast cancer (BC) [3]. However, the composition of immune cells within the TME and their interaction with tumor cells can directly impact immunotherapy efficacy. Moreover, a growing body of evidence suggests a link between antitumor immune responses and the prognosis of cancer patients [4,5,6]. Therefore, the cancer immunology research field holds promising value for the discovery of prognostic and predictive biomarkers, as well as for the improvement of existing strategies and the development of new immunotherapy strategies.

However, current knowledge of the tumor immune surveillance process mostly derives from analyses of primary tumor tissues, which cannot yet capture the dynamic alterations of the tumor immune crosstalk or identify specific immune patterns promoting disease progression in real time. On the other hand, local immune responses within the TME require continuous communication with the periphery. Therefore, the peripheral tissues and peripheral blood (PB) have gained significant attention in terms of exploring systemic immune responses in cancer patients [7]. To this end, analyses of the peripheral blood mononuclear cells (PBMCs), which include the key immune cell subsets circulating in the PB, are increasingly utilized to identify immune perturbations in cancer patients [8,9]. Moreover, circulating tumor cells (CTCs) can be identified in the PB of patients with solid tumors and represent a marker of metastatic dissemination and poor prognosis in several types of cancer, including BC [10]. CTCs adapt different immune evasion mechanisms in order to survive within the hostile environment of the PB, and their phenotypic analysis can uncover immune suppression mechanisms operating in real time [11]. In this context, we have previously reported the expression of different immunomodulatory molecules, such as PD-L1, CD47, TLR4, and pSTAT3, on both CTCs and PBMCs of BC patients, and their association with disease stage, response to treatment, and patient prognosis [12,13]. These studies suggest that the phenotypic analysis of CTCs and PBMCs may serve as a useful liquid biopsy tool to explore antitumor immune responses in real time and to identify promising biomarkers.

In the current study, we investigated the expression of two other important immunomodulatory molecules, the transmembrane receptor Fas (CD95/APO-1) and its ligand, FasL (CD95L), in the PB of patients with metastatic BC. The Fas/FasL pathway is a major regulator of caspase-dependent apoptosis in several physiological and pathological conditions, with a critical role in immune homeostasis, inflammation, and cancer immunity [14]. The Fas/FasL axis is frequently used by immune cells to trigger the apoptotic signaling in tumor cells, and vice versa, by tumor cells to induce the apoptosis of immune cells, which is known as the “Fas counterattack” [15,16]. Moreover, the Fas/FasL signaling on tumor cells can significantly increase their metastatic capacity via the induction of cancer stem cell (CSC) and epithelial to mesenchymal transition (EMT) properties [17,18,19,20]. Thus, it could also have a role in the biology and metastatic potential of CTCs. Numerous studies demonstrate the expression of Fas and FasL on cancer tissues, with significant prognostic relevance for patients with different malignancies, including BC [19,21,22,23]. However, the role of the Fas/FasL pathway in the periphery is largely unexplored. Based on the above, we herein assessed for the first time the expression of Fas and FasL on CTCs and circulating immune cells (PBMCs) in the PB of BC patients to investigate their potential role in the peripheral immune response and prognosis of BC.

## 2. Materials and Methods

### 2.1. Patients

The current study included 98 patients with metastatic BC who received first-line treatment at the Department of Medical Oncology, University General Hospital of Heraklion, Greece, between 2011 and 2017. Clinical characteristics and follow-up information were prospectively collected. Patients with secondary malignancies or incomplete clinicopathological data were excluded from the study. Consecutive patients with available blood samples who met the following criteria were included: pathologically diagnosed BC, age over 18 years old, ability to provide written informed consent, and complete clinical and pathological data. One patient was excluded from the survival analysis due to death from anaphylaxis on treatment initiation.

PB samples were collected at the baseline before first-line treatment and were analyzed within 1–2 h at the Laboratory of Translational Oncology (LTO), University of Crete, Heraklion, Greece. To assess the specificity of the CTC detection approach, PB samples were also collected from 20 healthy volunteers who had provided informed consent to participate in the study.

### 2.2. Cell Lines

Cytospins of representative BC cell lines (SKBR-3, MCF-7, and MDA.MB.231) and lung cancer cell lines (A549 and H1975) were analyzed to identify the optimal control for Fas and FasL expression. High expression of both Fas and FasL was evident in the H1975 cell line only (Figure S1), which was therefore selected to optimize the protocol of triple staining for cytokeratins (CK)/Fas/FasL/dapi and to serve as controls for patient samples.

The H1975 lung carcinoma cell line was obtained from the American Type Culture Collection (ATCC). Cells were cultured in Gibco™ D-MEM 4.5 g/L D-glucose (Thermo Fisher Scientific, Waltham, MA, USA) supplemented with 10% fetal bovine serum (FBS) (Thermo Fisher Scientific) and 1% penicillin/streptomycin (P/S) (Thermo Fisher Scientific), as previously described [24]. Cells were maintained in a humidified atmosphere of 5% CO^2−^ 95% air at 37 °C, and sub-cultivation was performed using ethylenediaminetetraacetic acid (EDTA)/Trypsin 0.25% (GIBCO-BRL). Following mycoplasma testing using the MycoAlertTM assay, cell cytospins were prepared and stored at −80 °C until use.

### 2.3. PBMC and CTC Enrichment

PB (10mL in EDTA) was obtained at the middle of vein puncture after the first 5 mL were discarded in order to avoid contamination with epithelial cells from the skin. The isolation of PBMCs, comprising a population of CTCs, was performed as described in our previous reports [12,13,24,25]. Briefly, Ficoll–Hypaque density-gradient (d = 1.077 g/mL) (Merck KGaA, Darmstadt, Germany) centrifugation was performed at 650× *g* for 30 min, and cells were washed twice with phosphate-buffered saline (PBS). Cytospins of 500,000 cells were prepared and stored at −80 °C until use.

### 2.4. Immunofluorescence (IF)

A total of 2 × 10^6^ PBMCs per patient were stained using two distinct IF assays: CK/Fas/FasL/dapi and CK/CD45/dapi (two slides each; total number of slides: *n* = 392).

The CK/Fas/FasL/dapi IF staining was developed using H1975 cytospins as controls for Fas and FasL expression. The optimized protocol included a fixation step with PBS/FA 3.7% for 15 min, RT; permeabilization with PBS/Triton X-100 0.1% for 10 min, RT; and blocking with PBS/FBS 5% for 2 h, RT. A primary antibody cocktail was prepared for overnight incubation at 4 °C, including two different Alexa Fluor 488-conjugated mouse anti-CK antibodies [Clones: AE1/AE3 (1:100) (Thermo Fisher Scientific) and C11 (1:200) (Novus Biologicals, LLC, Centennial, CO, USA)], along with a DyLight 650-conjugated mouse anti-Fas antibody (1:100) (Novus Biologicals) and rabbit anti-FasL antibody (1:75) (Purified MaxPab, Abnova GmbH, Heidelberg, Germany). Following the overnight incubation, Alexa fluor 555 Anti-Rabbit (1:300) was added for 45 min at RT as a secondary antibody for FasL. DAPI antifade (Invitrogen, Carlsbad, CA, USA) was added to identify cell nuclei.

The CK/CD45/dapi staining was performed using the above two Alexa Fluor 488-conjugated mouse anti-CK antibodies [(Clones: AE1/AE3 (1:100), and C11 (1:200)] and a rabbit anti-CD45 antibody (1:100) (H-230; Santa Cruz Biotechnology, Inc. Dallas, TX, USA; sc-25590), as previously described [12,13,24,25]. PBMC cytospins preparations from healthy volunteers (*n* = 20) were also stained for CK/CD45/dapi to evaluate the specificity of the CTC detection approach.

### 2.5. Assessment of Fas/FasL Expression on CTCs and PBMCs

Samples were stained for (a) CK/CD45/dapi to confirm that detectable CK+ cells are negative for CD45 and thus can be defined as CTCs and for (b) CK/Fas/FasL/dapi to assess Fas and FasL expression on CTCs (CK+ cells) and PBMCs (CK- cells).

The analysis was performed by two individual observers (E.P. and S.C.), who were blinded to each other’s findings and patients’ clinical data. The detection of at least one intact, nucleated cell, which was positive for CK, was used to define patient positivity for CTCs, as described in our previous reports [12,13,25].

Fas and FasL expression levels were first measured in cytospins of H1975 cells, which were included as controls in the IF stainings of patient samples. Briefly, the intensity of each marker was measured using the Ariol microscopy system (Genetix, New Milton, UK), with intensity values representing the exposure time required for detection of the fluorescent signal. As described in our previous reports, the lowest value determined in the negative control (one for each marker, including the secondary IgG isotype antibody only) represented the cut-off used to discriminate positive from negative expression [12,13,25].

Fas/FasL expression levels were then measured on single CTCs and PBMCs and were accordingly characterized as negative or positive, by using the previously defined cut-offs in the H1975 control cells. The detection of at least one CTC, positive for Fas or FasL, was used to define patient positivity for the respective marker [12,13,25]. The expression of two markers was also evaluated among 1.000 PBMCs in randomly selected microscopy vision fields; the detection of any (≥0%) expression was used to define PBMC positivity as previously described [12,13].

### 2.6. Statistical Analysis

A Fisher’s exact test was used to assess the possible correlation between different CTC and PBMC parameters and patient and disease characteristics. The progression-free survival (PFS) was calculated from the initiation of first-line treatment until the date of disease progression or death from any cause, whereas overall survival (OS) was calculated from the start of first-line treatment to death from any cause. Kaplan–Meier and Cox regression analyses were used to evaluate possible associations between different variables and survival measures. Parameters with statistical significance in univariate Cox regression analysis were subsequently included in a multivariate Cox proportional hazards regression model. All statistical analyses were performed using IBM SPSS Statistics version 20, and *p* values were considered statistically significant at the *p* < 0.05 level.

## 3. Results

### 3.1. Patient and Disease Characteristics

The patient and disease characteristics are summarized in Table 1. Among the 97 patients who were eligible for survival analysis, 82 had progressed (median PFS: 12.8 months; 95% CI: 11.1–14.6), and 75 had died (median OS: 32.7 months; 95%CI: 26.6–38.9) at the time of analysis.

### 3.2. CTC Detection and Characterization According to Fas/FasL Expression

The CK/CD45/dapi staining of blood samples from 98 BC patients and 20 healthy volunteers revealed the detection of CK+/CD45- CTCs in 21/98 (21.8%) of patients but not in healthy donors. Moreover, no CK+/CD45+ cells were detected in either patients’ or healthy donors’ samples, thus confirming the high specificity of the anti-CK antibodies used here and in our previous studies [12,13,24,25]. Additional staining for CK/Fas/FasL/dapi revealed the detection of 70 CK+ CTCs in 26/98 (26.5%) patients (mean no of CTCs per patient: *n* = 2). Consequently, the assessment of Fas/FasL expression on CTCs was feasible among these 26 CTC-positive patients.

**Figure 1:**
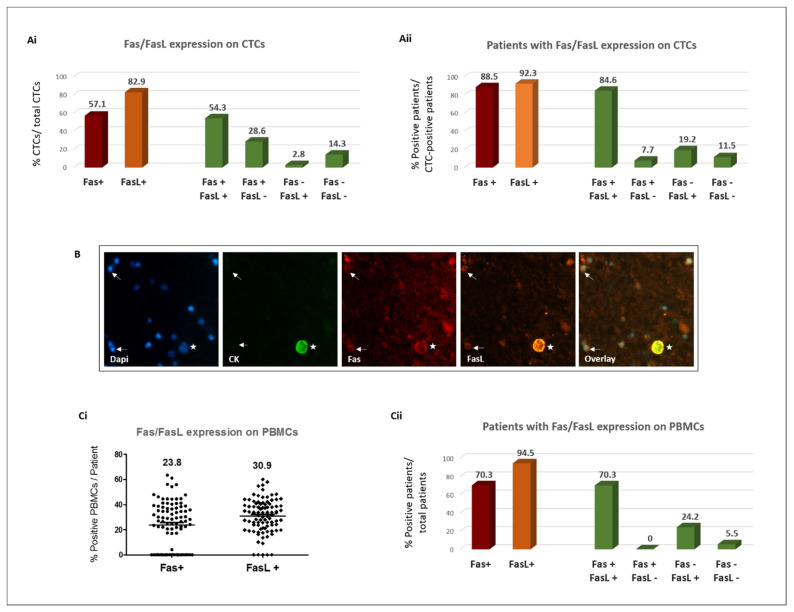
Distribution of Fas and FasL expression on circulating tumor cells (CTCs) and peripheral blood mononuclear cells (PBMCs) of patients with BC. Frequency of distinct CTC phenotypes among total CTCs (**Ai**) and among CTC-positive patients (**Aii**) (*n* = 26 patients). (**B**) Representative fluorescence microscopy image of a BC patient’s blood sample; CK (green), Fas (red), FasL (orange), and dapi (blue for cell nuclei), ×400. Star: A Fas+/FasL+ CTC (CK+ cell), white arrows: Fas+/FasL+ PBMCs (CK- cells). Distribution of Fas/FasL expression on PBMCs; (**Ci**) percentage of positive PBMCs per patient; lines represent mean values, (**Cii**) frequency of distinct PBMC phenotypes among total patients (*n* = 98 patients).

The results show that Fas and FasL are frequently expressed on the CTCs of patients with metastatic BC. Specifically, Fas+ CTCs and FasL+ CTCs were identified in 88.5% and 92.3% of CTC-positive patients, respectively, and represented 57.1% and 82.9% of total CTCs (Figure 1Ai–Aii). Co-expression analysis on single cells revealed the detection of Fas+/FasL+ CTCs in 84.6% of CTC-positive patients, representing 54.2% of total CTCs (Figure 1Ai–Aii). A representative image of a Fas+/FasL+ CTC is shown in Figure 1B (indicated by a star).

### 3.3. Evaluation of Fas/FasL Expression on PBMCs and Correlation with CTC Phenotype

Fas and FasL were expressed on PBMCs in 70.3% and 94.5% of patients, respectively, with a mean percentage of positive PBMCs per patient: 23.8 ± 1.9% and 30.9 ± 1.4%, respectively (Figure 1Ci). The combined analysis of the two markers revealed that the majority of patients (70.3%) harbored the Fas+/FasL+ PBMC phenotype, whereas the Fas-/FasL+ and Fas-/FasL- phenotypes were evident in 24.2% and 5.5% of patients, respectively (Figure 1Cii); interestingly, no Fas+/FasL- PBMCs were detected in any patient.

No correlation was shown between the phenotype of PBMCs and the detection of CTCs; however, a significant association was demonstrated between the PBMC and CTC phenotypes. Specifically, Fas+/FasL+ CTCs correlated with Fas+/FasL+ PBMCs (*p* = 0.002 Fisher’s exact test, Table S1); also, patients with Fas-/FasL+ CTCs had Fas-/FasL+ PBMCs only, while one patient with Fas-/FasL- CTCs had Fas-/FasL- PBMCs only (*p* = 0.002, Fisher’s exact test, Table S1).

### 3.4. Correlation of CTC and PBMC Parameters with Patient and Disease Characteristics

No association was shown between the detection or phenotype of CTCs and age, menopausal status, histology and molecular subtype of the tumor, or the number of disease sites. However, significant associations were confirmed between CTCs and the organ of secondary metastasis; specifically, patients with bone metastases more frequently harbored CTCs (in 39.5% vs. 17.2% of patients, *p* = 0.015), and more particularly Fas+ CTCs (34.2% vs. 15.5%, respectively *p* = 0.033) and FasL+ CTCs (36.8% vs. 15.5%, respectively *p* = 0.017), as compared to those without bone metastases.

No association was shown between PBMC phenotypes and patient and disease characteristics.

### 3.5. Correlation of CTC and PBMC Parameters with Survival Measures

#### 3.5.1. CTC Detection and Phenotype

**Figure 2:**
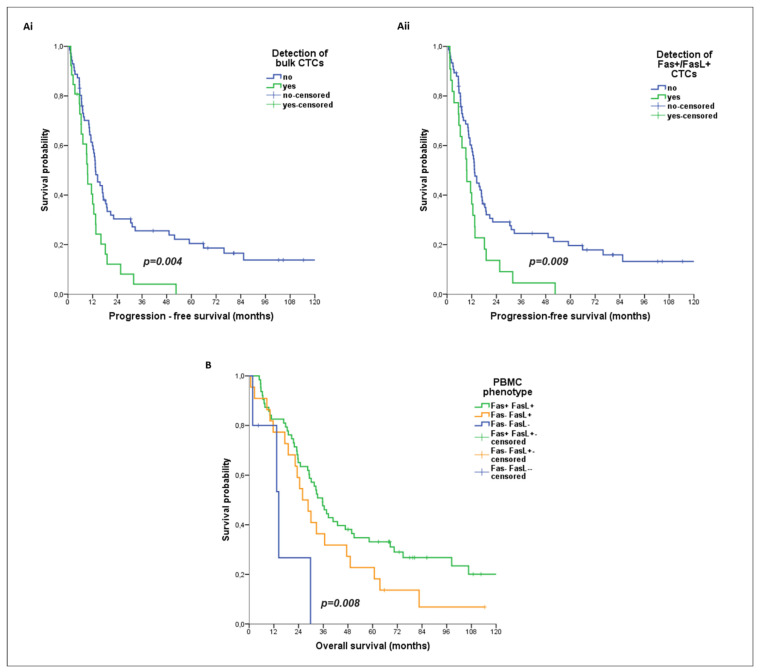
**Prognostic relevance of CTC and PBMC phenotypes in patients with metastatic BC.** Kaplan–Meier plots for progression-free survival (PFS) based on the detection of CTCs (**Ai**) and particularly Fas+/FasL+ CTCs (**Aii**). (**B**) Kaplan–Meier plots for overall survival (OS) based on the detection of all distinct PBMC phenotypes (**B**). Log rank test was used for *p* value calculations (*n* = 97 patients).

Kaplan–Meier analysis revealed a reduced PFS among patients with detectable CTCs as compared to CTC-negative patients (median PFS: 9.5 vs. 13.4 months; *p* = 0.004) (Figure 2Ai), and specifically among those harboring the Fas+/FasL+ CTC subpopulation (median PFS: 9.5 vs. 13.4 months; *p* = 0.009) (Figure 2Aii).

In univariate analysis for PFS, a high risk for disease progression was demonstrated among patients with detectable CTCs (HR: 1.987; 95% CI: 1.226–3.220; *p* = 0.005) and Fas+/FasL+ CTCs (HR: 1.917; 95% CI: 1.165–3.154; *p* = 0.010), as well as those with triple negative subtype (HR: 2.447; 95% CI: 1.154–5.188; *p* = 0.020) (Table 2). In multivariate analysis for PFS, triple negative subtype (HR: 2.397; 95% CI: 1.129–5.089; *p* = 0.023) and the detection of CTCs (HR: 1.922; 95% CI: 1.169–3.160; *p* = 0.010) and Fas+/FasL+ CTCs (HR: 1.839; 1.107–3.055; *p* = 0.019) emerged as independent factors predicting the high risk for disease progression (Table 2).

No association was shown between CTC parameters and OS rates or the risk of death (Table 2).

#### 3.5.2. PBMC Phenotype

There was no association between PBMC parameters and PFS or the risk for progression. However, Kaplan–Meier analysis revealed an increased OS among patients harboring Fas+/FasL+ PBMCs, as compared to those with Fas-/FasL+ PBMCs and Fas-/FasL- PBMCs (median OS: 35.7 vs. 25.9 vs. 14.4 months; *p* = 0.008) (Figure 2B).

In a univariate analysis for OS, a high risk for death was demonstrated among patients with age above the median (HR: 1.800; 95% CI: 1.136–2.852; *p* = 0.012) and more than two disease sites (HR: 1.772; 95% CI: 1.102–2.849; *p* = 0.018). On the contrary, the Fas+/FasL+ PBMC expression pattern was correlated with a lower risk for death (HR: 0.234; 95% CI: 0.081–0.670; *p* = 0.007) (Table 2). Multivariate analysis for OS confirmed that age above median (HR: 2.071; 95% CI: 1.260–3.405; *p* = 0.004) and high number of disease sites (HR: 2.431; 95% CI: 1.451–4.073; *p* = 0.001) independently predicted for high risk for death, whereas the Fas+/FasL+ PBMC phenotype emerged as independent factor predicting a lower risk for death (HR: 0.161; 95% CI: 0.054–0.485; *p* = 0.001) (Table 2).

## 4. Discussion

The Fas/FasL system represents one of the major apoptotic pathways that regulate numerous physiological and pathological processes mediated through programmed cell death, which also has a critical role in anticancer immunity. Accumulating evidence in animal models and human cancer tissues show that the Fas/FasL pathway can be exploited by immune cells and tumor cells, exhibiting both antitumor and tumor-promoting effects; however, very limited data exist on the role of this pathway in the periphery. In the current study, we investigated for the first time the expression of Fas and FasL on the tumor (CTCs) and immune (PBMCs) cell compartments in the PB of patients with metastatic BC. The results showed that Fas and FasL were both expressed in the vast majority of CTCs and were also frequently expressed on PBMCs from BC patients. Notably, the detection of CTCs, particularly Fas/FasL co-expressing CTCs, emerged as an adverse prognostic factor, while in contrast, Fas/FasL expression on PBMCs was associated with favorable outcomes of patients with metastatic BC.

Fas receptor is a member of the tumor necrosis factor receptor (TNF-R) subfamily that, following interaction with its ligand, FasL, triggers a signal transduction pathway leading to apoptosis [14]. The Fas/FasL system is frequently exploited by tumor cells to evade the host’s immune response. During the so-called “Fas counterattack”, tumor cells up-regulate FasL to induce apoptosis in Fas-expressing immune cells, thus leading to immune suppression [15,16]. Vice versa, Fas-expressing tumor cells may be vulnerable to apoptosis evoked by FasL-positive tumor-specific immune cells. However, the Fas/FasL-mediated apoptotic signal is often defective on cancer cells, thus triggering pro-tumorigenic cellular outcomes rather than apoptosis. In this context, Fas activation on tumor cells can induce the NF-κB, ERK1/2-MAPK, and PI3K/Akt pathways, thus promoting their migration and acquisition of EMT and CSC-like properties [17,18,19,20,26]. Numerous studies have reported the expression of Fas and FasL in cancer cell lines and tumor tissues [22,27,28,29]. However, there are no data showing their expression at the CTC level so far. We herein showed for the first time that Fas and FasL were frequently expressed and co-expressed on BC patients’ CTCs, suggesting that the Fas/FasL system may also operate on cancer cells in the periphery. Accordingly, we have previously shown that CTCs from BC patients often display CSC-like and EMT-like features [25,30,31] and express putative immune checkpoints with a key role in tumor immune evasion [12,13]. The current study significantly adds to the current knowledge of the mechanisms employed by CTCs, possibly facilitating their immune escape, survival, and migration through the hostile blood environment.

Notably, the detection of CTCs, particularly of Fas/FasL-co-expressing CTCs, emerged as a marker for reduced PFS and an independent factor predicting the high risk for disease progression in patients with metastatic BC. Although these findings should be interpreted with caution due to the low number of patients analyzed, they are in line with the acknowledged role of the Fas/FasL pathway in driving tumor immune evasion, metastasis, and chemoresistance [19,32,33,34]. Even though controversial data exist on the prognostic role of Fas expression on cancer tissues [19,21,35], numerous studies converge on the adverse prognostic value of tumoral FasL expression in breast, lung, esophageal, and colorectal cancer tissues [22,23,36]. Importantly, Fas and FasL can often be co-expressed on individual tumor cells [37,38,39], which has been shown to inactivate the downstream apoptotic signaling, thus protecting cells from apoptotic death [27,40]. This further supports our finding that Fas+/FasL+ CTCs represent the only CTC fraction with adverse prognostic value for BC patients. We could, therefore, hypothesize that CTCs co-expressing Fas and FasL may constitute an aggressive subset that would be resistant to apoptosis evoked by FasL-expressing tumor-specific immune cells and, at the same time, could induce apoptosis in Fas-expressing immune cells; however, this hypothesis lacks functional validation. Nevertheless, the current findings corroborate our previous studies, showing that CTC detection and phenotyping can provide valuable prognostic information for BC patients [12,13,25,30,41].

Regarding the distribution of Fas/FasL on immune cells, both molecules can be expressed by CD4+ and CD8+ T cells, B cells, natural killer (NK) cells, dendritic cells (DCs), and macrophages, with most studies pointing toward a dual role of Fas/FasL signaling in peripheral immune tolerance [42,43,44]. Specifically in cancer, it is widely accepted that the Fas/FasL system plays a critical role in the dynamic tumor immune crosstalk [16,45,46]. Most studies of human tumor tissues have focused on the association between Fas/FasL expression on cancer cells and the levels of surrounding tumor-infiltrating lymphocytes (TILs) or apoptotic TILs [47,48]. Nevertheless, there is important evidence showing that Fas and FasL can be frequently expressed on TILs [28,49,50], as well as on specific immune cell subsets circulating in the PB of patients with solid tumors [51,52]. In the current study, we analyzed for the first time the expression of Fas/FasL on bulk PBMCs, which encompass the entire immune cell compartment circulating in the PB. We showed that both Fas and FasL were commonly expressed on BC patients’ PBMCs. In line with our findings, a higher incidence of circulating Fas+ cytotoxic T-cells has been shown among patients with early BC or hepatocellular carcinoma (HCC) as compared to healthy donors [51,52]. Moreover, a frequent expression of Fas and FasL has been reported on TILs from BC tissues [28]. Most importantly, in the current study, positivity for both Fas/FasL on PBMCs emerged as a favorable prognostic marker associated with improved OS rates and an independent factor predicting the low risk for death in patients with metastatic BC. This is the first study showing the prognostic value of Fas/FasL expression on peripheral immune cells in BC. We have previously used the same PBMC phenotyping approach to uncover immune checkpoint expression patterns with prognostic and/or predictive relevance in BC [12,13] and small cell lung cancer (SCLC) (unpublished observations). Taken together, our studies suggest that, besides CTCs, PBMCs also hold a promising role as biomarkers for patients with solid tumors, and their analysis may add significant value to future liquid biopsy-based studies.

The above findings collectively suggest that Fas/FasL co-expression displays an adverse prognostic role when detected on CTCs and a favorable role when identified on the PBMCs of BC patients. As already mentioned above, the parallel expression of Fas and FasL confers cells with resistance to extrinsic Fas/FasL-mediated apoptosis [40], which could provide an explanation for the opposite prognostic value revealed among the tumor and immune cell compartments. In the study by Gruber I. et al., CTC detection was associated with increased levels of circulating Fas+ T-helper cells in the PB of patients with early-stage BC [53]. Here, we did not observe any association between CTC detection and Fas/FasL phenotype on PBMCs; however, a positive correlation was shown in the Fas/FasL expression patterns among the CTC and PBMC compartments. This observation could indicate an interrelated activation of the Fas/FasL system in tumor and immune cells in the periphery that might reflect a broad activation of peripheral antitumor immune response. The analysis of additional immune checkpoints would help to define the interplay of Fas/FasL with other immune-related pathways and to understand their role in the overall immune response status in patient individuals. Moreover, future studies could identify, through gene expression profiling, transcription factors and signaling pathways responsible for modulating Fas and FasL levels on CTCs and PBMCs.

The lack of functional assays is an important limitation of our study. In vitro co-cultures and CTC-derived xenografts (CDXs) would be required to define the distinct roles of Fas/FasL pathway among CTCs and circulating immune cells and its impact on the tumor-immune crosstalk in the PB of BC patients. Moreover, in vitro models mimicking the TME, as well as analyses of matched primary tumor tissues, would provide important insights into how the tumor milieu might influence the expression of Fas/FasL in the periphery. Also, consecutive blood samples from these patients were not available for longitudinal assessment of the Fas/FasL expression status. All these issues could be addressed in a future prospective study. Another potential limitation of the current study might be the use of ficoll density gradient centrifugation for CTC enrichment since this approach provides low CTC positivity rates as compared to currently available automated enrichment platforms [24]. Although our previous studies converge on the prognostic value of ficoll-enriched CTCs for BC patients [12,13,25,30,41], the availability of high CTC counts for downstream analysis would help to better define the incidence and role of Fas/FasL expression on CTCs. Moreover, considering that PBMC analysis at the single-cell level is time-consuming, our methodology has the limitation of analyzing low numbers of PBMCs (approximately 1000 cells per sample), thus making the assessment of rarely expressed molecules challenging and questionable. Nevertheless, we have repeatedly used the current approach for single PBMC phenotyping to identify clinically relevant PBMC expression patterns in patients with different malignancies [12,13]. Moreover, the analysis of the whole PBMC compartment, rather than specific immune cell subsets, can be used to evaluate the overall expression status of immune checkpoints in circulating immune cells, with reduced cost and technical requirements. In addition, the methodology used here allows the combined assessment of CTCs and PBMCs within the same sample, thus further reducing the overall cost and time of analysis.

To summarize, we here show, for the first time, that Fas and FasL are frequently expressed on BC patients’ CTCs and PBMCs, providing important prognostic information for patient outcomes. Our previous and present findings suggest that phenotypic analyses of CTCs and PBMCs can capture the dynamic changes in tumor and immune cell expression profiles, thus informing on the status of different prognostic and predictive biomarkers in real time [12,13]. To date, different therapeutic strategies targeting the Fas/FasL system have been developed and seem to efficiently prevent the metastatic progression of tumors [54,55,56,57]. Recent data suggest that the induction of apoptosis pathways via targeting the CD74/Fas and CD74-AKT axes could be a promising treatment strategy for triple negative BC [58]. Moreover, preclinical evidence derived from a TME-mimicking tissue culture system enriched with Fas molecules and PBMCs treated with an anti-Fas monoclonal antibody (mAb) revealed that blocking the Fas/FasL pathway could significantly increase apoptosis and reduce stemness of BC cells [59]. Studies in larger cohorts of patients would help to understand the prognostic and therapeutic opportunities emerging from the assessment of the Fas/FasL pathway in liquid biopsies in BC.

## 5. Conclusions

The current study provides, for the first time, evidence on the expression of Fas and FasL on CTCs and PBMCs, with significant prognostic value for patients with metastatic BC. These findings suggest a potential role of the Fas/FasL system in the peripheral antitumor response and metastatic progression of BC. In line with our previous studies, the value of PBMCs as a promising liquid biopsy biomarker in cancer is further highlighted. The prognostic and therapeutic implications of these findings merit further investigation.

## Figures and Tables

**Table 1:** Patient and disease characteristics of patients with metastatic breast cancer (BC).

Metastatic BC Patients (*n* = 98)	*n* (%)
Age, years; median (range)	59 (29–84)
Menopausal status	
Pre-menopausal	27 (27.6)
Post-menopausal	69 (70.4)
Unknown	2 (2)
Histology	
Ductal	81 (82.7)
Lobular	9 (9.2)
Mixed	5 (5.1)
Unknown	3 (3.1)
Stage at diagnosis	
I–III	70 (71.4)
IV	28 (28.6)
Subtype	
ER+ and/or PR+/HER2-	63 (64.3)
HER2+	23 (23.5)
Triple negative	12 (12.2)
Metastases type	
Visceral only	27 (27.6)
Non-visceral only	29 (29.6)
Both	39 (39.8)
Unknown	3 (3.1)
Disease sites	
1–2	62 (63.3)
>2	33 (33.7)
Unknown	3 (3.1)
Prior adjuvant treatment	
Chemotherapy	49 (50)
Hormone therapy	16 (16.3)
No	30 (30.6)
Unknown	3 (3.1)
First-line treatment	
Chemotherapy	86 (87.8)
Hormone therapy	12 (12.2)
Response to treatment at first evaluation	
Partial response (PR)	38 (38.8)
Stable disease (SD)	34 (34.7)
Progressive disease (PD)	9 (19.4)
Non-evaluable (NE)	7 (7)

**Table 2:** Univariate and multivariate Cox-regression analysis for PFS and OS among patients with metastatic BC.Cox Regression Analysis.

	Progression-Free Survival (PFS)				Overall Survival (OS)			
	Univariate		Multivariate		Univariate		Multivariate	
Covariates	HR (95% CI)	*p* Value	HR (95% CI)	*p* Value	HR (95% CI)	*p* Value	HR (95% CI)	*p* Value
Age (≥59 vs. <59 years)	1.281 (0.823–1.994)	0.273			1.800 (1.136–2.852)	0.012 *	2.071 (1.260–3.405)	0.004 *
Menopausal Status (post vs. pre)	1.283 (0.606–2.713)	0.515						
Performance Status (2–3 vs. 1)	2.142 (0.660–6.950)	0.205			2.500 (0.773–8.082)	0.126		
Histology								
Mixed	Reference				Reference			
Ductal	1.156 (0.421–3.174)	0.778			0.782 (0.283–2.160)	0.635		
Lobular	1.691 (0.505–5.664)	0.395			1.357 (0.417–4.420)	0.612		
Stage at diagnosis (I-III vs. IV)	1.492 (0.912–2.440)	0.111			1.476 (0.881–2.470)	0.139		
Molecular subtype of tumor								
HER2+	Reference		Reference		Reference			
ER+ and/or PR+/HER2-	1.635 (0.940–2.842)	0.081	1.424 (0.807–2.513)	0.222	1.625 (0.910–2.902)	0.101		
Triple negative	2.447 (1.154–5.188)	0.020 *	2.397 (1.129–5.089)	0.023 *	1.7.57 (0.786–3.930)	0.170		
Visceral metastases (yes vs. no)	1.091 (0.683–1.744)	0.716			1.101 (0.666–1.818)	0.708		
No. of disease sites (>2 vs. ≤2)	1.274 (0.802–2.024)	0.305			1.772 (1.102–2.849)	0.018 *	2.431 (1.451–4.073)	0.001 *
CTC detection (yes vs. no)	1.987 (1.226–3.220)	0.005 *	1.922 (1.169–3.160)	0.010 *	1.343 (0.815–2.213)	0.247		
CTC phenotype								
Fas+/FasL+ CTCs	1.917 (1.165–3.154)	0.010 *	1.839 (1.107–3.055)	0.019 *	1.374 (0.814–2.320)	0.234		
Fas+ or FasL+ CTCs	1.875 (0.813–4.326)	0.140			1.320 (0.572–3.049)	0.515		
Fas-/FasL- CTCs	2.522 (0.607–10.485)	0.203			3.486 (0.838–14.503)	0.086		
PBMC phenotype								
Fas-/FasL- PBMCs	Reference				Reference		Reference	
Fas-/FasL+ PBMCs	0.675 (0.226–2.020)	0.483			0.357 (0.119–1.069)	0.066	0.269 (0.119–1.069)	0.024
Fas+/FasL+ PBMCs	0.678 (0.243–1.890)	0.458			0.234 (0.081–0.670)	0.007 *	0.161 (0.081–0.670)	0.001 *

* Statistical significance at the *p* < 0.05 level. Only variables showing statistical significance in univariate analysis were subsequently included in multivariate analysis (molecular subtype of tumor, the detection of CTCs or Fas+/FasL+ CTCs were tested for PFS, whereas age, number of disease sites, and the PBMC co-expression pattern were tested for OS) (no of patients: *n* = 97).

## Data Availability

The original contributions presented in the study are included in the article and supplementary material; further inquiries can be directed to the corresponding author.

## Supplementary Materials

The following supporting information can be downloaded at https://www.mdpi.com/article/10.3390/cancers16172927/s1, Figure S1: Distribution of cytokeratins (CK), Fas, and FasL expression in breast and lung cancer cell lines. Representative immunofluorescence microscopy images of CK (green), Fas (red), and FasL (orange) expression and dapi (cell nuclei; blue) among breast (SKBR3, MCF7, and MDA.MB.231) and lung cancer cell lines (A549 and H1975); Ariol system (×400).; Table S1: Correlation between CTC and PBMC phenotypes (Crosstab table). CTC-positive patients were included only (*n* = 26 patients); values represent patient numbers within each group. Fisher’s exact test; *p* = 0.002.
