# Supplementary material for: Prognostic Value of Fas/Fas Ligand Expression on Circulating Tumor Cells (CTCs) and Immune Cells in the Peripheral Blood of Patients with Metastatic Breast Cancer
Source: Cancers (Basel). 2024 Aug 23;16(17):2927. doi: 10.3390/cancers16172927 (PMC11393959; doi:10.3390/cancers16172927)
Supplement: Supplementary file 1 [file cancers-16-02927-s001.zip › Table S1.pdf]

Table S1. Correlation between CTC and PBMC phenotypes (Crosstab table).

Crosstab (No of patients)	PBMC phenotype			
CTC phenotype	Fas+/FasL+	Fas-/FasL+	Fas-/FasL-	sum
Fas+/FasL+	18	1	2	21
Fas+/FasL-	1	0	0	1
Fas-/FasL+	0	3	0	3
Fas-/FasL-	0	0	1	1
sum	19	4	3	26

CTC-positive patients were included only (n=26 patients); values represent patient numbers within each group. Fisher's exact test; p=0.002.
