# Supplementary figures and images for: Prognostic Value of Fas/Fas Ligand Expression on Circulating Tumor Cells (CTCs) and Immune Cells in the Peripheral Blood of Patients with Metastatic Breast Cancer
Source: Cancers (Basel). 2024 Aug 23;16(17):2927. doi: 10.3390/cancers16172927 (PMC11393959; doi:10.3390/cancers16172927)

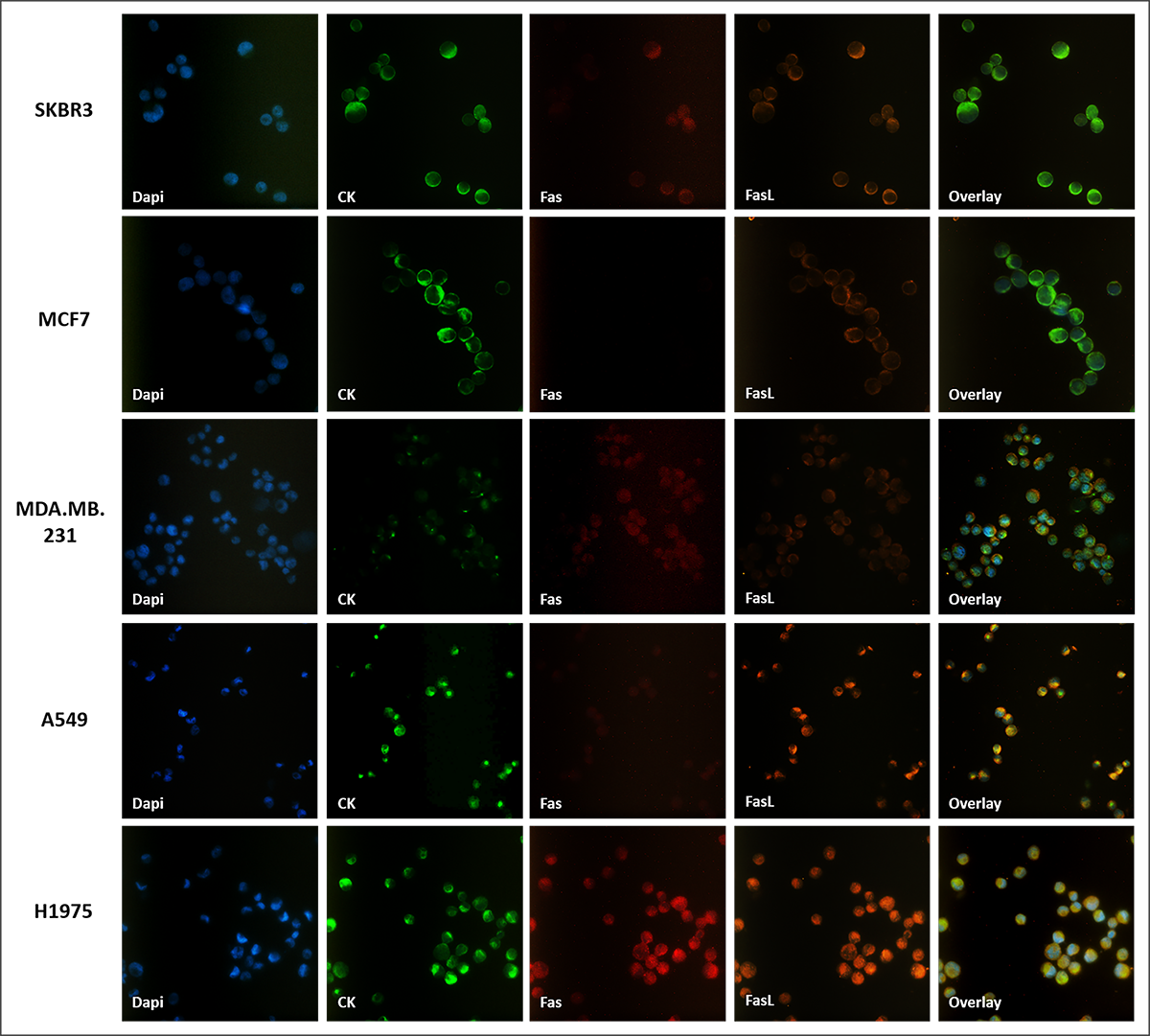

Supplement: Supplementary file 1 [file cancers-16-02927-s001.zip › Figure S1.tif]
